# Identifying and Implementing Strategies to Reduce the Risk of Self-Contamination of Health Care Workers Caused by Doffing of Personal Protective Equipment During the COVID-19 Pandemic

**DOI:** 10.1017/dmp.2020.396

**Published:** 2020-10-22

**Authors:** Sai Saran, Mohan Gurjar, Atul Garg

**Affiliations:** Department of Critical Care Medicine, Superspeciality Cancer Institute and Hospital, Lucknow, Uttar Pradesh, India; Department of Critical Care Medicine, Sanjay Gandhi Post Graduate Institute of Medical Sciences (SGPGIMS), Lucknow, Uttar Pradesh, India; Department of Microbiology, Sanjay Gandhi Post Graduate Institute of Medical Sciences (SGPGIMS), Lucknow, Uttar Pradesh, India

**Keywords:** doffing, health care worker, infection control, personal protective equipment

## Abstract

During the current coronavirus disease (COVID-19) pandemic, it is estimated that tens of thousands of health care workers have been infected. The doffing of personal protective equipment (PPE) has been identified an important place and procedure that might influence the self-contamination of health care workers. More recent evidence suggests that, in addition to existing infection control standards, there is an urgent need for the incorporation of various recent information and advancements pertaining to structure and process to reduce the self-contamination of health care workers during the doffing of PPE.

In light of the current coronavirus disease (COVID-19) pandemic, health care facilities are faced with a shortage of health care workers (HCWs), a problem that is compounded by self-contamination of the HCWs, despite using personal protective equipment (PPE).^1,2^ During the current pandemic, it is estimated that tens of thousands of HCWs have been infected with COVID-19, worst cases being in Italy with up to 20% of HCWs infected.^1,2^ In the month of April 2020, there were many deaths of HCWs reported from various countries, including 27 in the United States, 106 in the United Kingdom, and 180 in Russia.^2^ In general, aerosol generating procedures like tracheal intubation, non-invasive ventilation, and tracheostomy are being considered as main risks of transmission of acute respiratory infection to HCWs. Transmission through contact of high touch surfaces and PPE can also account for a substantial proportion of such infections.^3-5^

The doffing of PPE has been identified an important place and procedure that might influence self-contamination of HCWs. High biological loads of the novel coronavirus had been detected in the air of patient care areas prone to crowding like toilet areas and protective apparel removal rooms (doffing zone) when measured using droplet digital PCR-based detection methods (ddPCR) in hospitals at Wuhan, China, the epicenter of COVID-19.^6^ Among the components of PPE, used gloves, gowns, and masks account for maximum bio-load, and these are routinely piled up in the doffing zone. This can generate high aerosol concentrations of the virus and poses a heightened risk of self-contamination.^4,7^ There are several factors that influence the rate of self-contamination of HCWs during doffing, for example, compliance of HCWs to using checklists and standardized operating procedures (SOPs), poor fitting of PPE, and damage to PPE during work due to either poor quality or shear stress or friction from various activities.^8,9^

This perspective provides available evidence of interventions and advancements to achieve the goal of the least possible self-contamination during doffing of PPE. These can be categorized as (1) physical space and air handling of the doffing zone; (2) biomedical waste (BMW) disposal; (3) PPE material; and (4) improving adherence to doffing protocols by HCWs (Table 1).

**TABLE 1:** Possible Interventions to Prevent Infection Among HCW During the Doffing of Personal Protective Equipment

Component	Intervention
Physical space and environmental related	Larger spaces should be divided into color-coded zones (dirty and clean) with extended lines. Appropriately place mirrors, handrails, or sitting arrangement. HVAC considerations: Provision of separate air handling unit with 100% fresh flow and minimal or no re-circulation 15–20 air changes per hour (to increase if re-circulation based system) Laminar flow pattern from clean to dirty zones applying “conforming to the path principle” Changing HEPA to ULPA filter, especially if re-circulation based system is used Frequent air sampling (ddPCR and IMA) Filter forensics at periodic time intervals Intelligent HVAC systems Install sterilizers and disinfectant devices that are effective and approved, that is, ultraviolet radiation device, photon mediated electrons, emitter devices, etc.
Biomedical waste disposal related	Use sensor-based or foot-operated large sized covered bins. Use high-level disinfectant (1% Virkon™, Oxivir^®^) in the bins themselves. Use biosafety cabinets with UV irradiation for piling up the PPE. Use conveyor belts to ensure that there is no piling up of the PPE in the doffing zone and their transport to terminal disinfection zone. Use artificial intelligence like automation about information and/or packing and movement of bins outside doffing zone once they’re filled.
PPE material related	Use fabric with less viral adherence and viricidal/property. Use fabric with adequate tensile and breaking strength. Use fluorescent tracer methods to detect self-contamination.
Human behavior related	Practice frequent training ensuring compliance to SOPs. Use tele-monitoring (2-way audio-visual). Use alarm system for ensuring adequate hand hygiene.

*Notes*: HVAC = heating, ventilation, and air conditioning; HEPA = high-efficiency particulate air (filter); ULPA = ultra-low penetration air (filter); ddPCR = droplet digital polymerase chain reaction; IMA = index of microbial air contamination; SOPs = Standard Operating Procedures; PPE = personal protective equipment; UV = ultraviolet (light).

## PHYSICAL SPACE AND ENVIRONMENT

There is a need for ensuring an optimal design and layout of the doffing zone, to ensure adequate space in order to dilute the biological infective load, as there can be more than 2 or 3 persons doffing simultaneously after a working shift.^10^ Apart from being separately located from the donning zone, this zone should have separate access points for HCWs, BMW disposal teams, and the monitor or the supervisor called the “dofficer,” preferably with real-time monitoring.^11^ This zone should have appropriately placed full-body length mirrors, handrails, or sitting arrangement aiding in the doffing of various components of the PPE.^10^

The doffing zone should be equipped with a separate air handling unit, negative pressurization, use of 100% fresh flow, a higher number (more than 20) of air changes per hour (ACH) with minimal or no re-circulation, and directed air flow from the clean to dirty zone (preferably laminar pattern) to reduce the concentration of the virus in the air.^8,12^ ACH can be decided based on the bio-load in the doffing zone, as even up to 100 were considered in walk-through screening centers for COVID-19, which can remove 99.99% of particles in the air within 3 to 5 minutes.^13,14^ High efficiency particulate air (HEPA) filters, which have 99.97% filtration efficiency at 0.3 µm sized particles, could be changed to ultra-low penetration air (ULPA) filters, which have 99.999% efficiency for up to 0.12 µm particles as the size of the SARS-CoV-2 virus ranges from 0.06–0.14 µm, especially if re-circulation based air distribution system is used.^14^ The heating, ventilation, and air conditioning (HVAC) system relies on “conforming to the path principle,” in order to avoid disturbances to the pattern of air flow by any additional or new equipment or personnel; once all the equipment is set up in the doffing zone and the path of movement of personnel is ascertained, the HVAC engineer should be consulted to look after and adjust the flow pattern and changes to be made accordingly.^12^ “Intelligent” HVAC systems which adjust their flow pattern as per the occupants, can also reduce the risk of contamination.^15^

The installation of approved environmental sterilizers, such as an ethylene-oxide (EO) gas aerator cabinet, and disinfectant devices, such as ultra violet germicidal irradiation that either emits continuous ultraviolet (UV-A or UV-C) light or a pulsed-xenon UV light system, photon mediated electron emitters, can reduce the viral load in the doffing zone.^16,17^ In addition, air purifiers (Medical UV air purifier) also have a role in such zones.^17^ The efficacy of the above measures should be ensured by frequent air sampling to estimate the viral load with ddPCR or with the index of microbial air contamination (IMA), apart from “filter forensics” at specified time intervals to understand the bioload in such zoning and planning interventions.^8,12^

## BIOMEDICAL WASTE DISPOSAL

There should be sensor-based or foot-operated large sized covered and clear demarcated bins using high level disinfectant such as 1% Virkon™, accelerated hydrogen peroxide based disinfectant (Oxivir®), when compared to those commonly used in disinfection of high touch surfaces (0.5–1% sodium hypochlorite).^17-19^ Separate labeled containers should be available to facilitate disinfection and maintenance of reusable components of PPE like powered air purifying respirators, other high-end respirators, and face shields.^19^ Recent advancements include the utility of biosafety cabinet-like systems, labeled “SAFETY,” in the screening zones of COVID-19; the same can be implied to the doffing zone in order to protect HCWs from self-contamination.^13^ Doffed PPE can be kept in biosafety cabinets (BSC Class 2 and above), in which the HEPA-filtered unidirectional air flow pattern is maintained. UV irradiation inside the BSC can further reduce the bioload before the BMW team collects them for disposal. The implementation of robot-based systems or conveyer belts to carry the potentially infective load (discarded PPE) from the doffing zone to the terminal disinfection zone offers an opportunity to reduce further exposure of HCWs involved in BMW management.^20^

## PERSONAL PROTECTIVE EQUIPMENT

In cases of PPE, while breathability and water vapor evaporation ensure comfort, the use of fabric with Class 3 exposure pressure and above ensures safety and carriage of the least possible biological load.^21^ A notable advancement in fabric design by a team from Indiana University, United States, has been the development of electroceutical fiber PPE, with alternating dots of silver and zinc batteries that can destabilize the virus by the creation of a weak electric field.^22^ This, and also the use of antimicrobial repellent finishes, can also reduce viral loads in the doffing zone. It is also essential to choose fabrics with adequate tensile and breaking strength, as damaged PPE incurs the risk of “pathogen carry over,” owing to increased microbial adhesion to damaged surfaces, especially during doffing.^7,23^ Identifying potential self-contamination on PPE, skin, and clothes worn by HCWs using fluorescent tracer methods offers another solution to mitigate this risk.^24^

## HUMAN BEHAVIOR AND COMPLIANCE TO PROTOCOLS

Adequate training and frequent monitoring of HCWs during donning and doffing can limit the transmission of infection, by reducing deviations from ideal techniques.^8,9,11^ Also, it is necessary to obtain frequent data regarding the facilitators (Is this the meaning you intended?like fitting of PPE) for improving the compliance, along with barriers like fogging, perspiration, as evaluated in a recent questionnaire-based study.^25^

The doffing zone needs extra monitoring by a “dofficer” in real time, in order to ensure the appropriate disposal of used PPE in compliance with the established SOPs in that particular facility.^11^ In a study of assessing common behavioral faults during doffing, Mumma et al. found that the most frequent steps leading to maximal non-compliance involved removing the outermost garment, boot covers, and respirator hood apart from hand hygiene.^26^

Ensuring hand hygiene with alcohol-based sanitizers and a minimum contact time of 20 seconds at every step of removal of each item of PPE is crucial.^7^ This could be ensured with the help of audible alarms or music for a requisite duration (20 seconds) after pressing the foot-operated sanitizer systems.^9^ The use of computer simulations ensuring the proper sequence of removal of PPE, incorporation of alarm systems in case the sequence is failed, and also the use of telecommunication (a supervisor giving instructions to the HCWs through audio-visual interface) can ensure a safe passage of the HCWs through the doffing zone, with minimal self- and cross-contamination risks.^10,19^

## CONCLUSION

Identifying and implementing strategies to reduce the chances of infection among HCWs are of utmost priority in any health care facility, especially in the time of the ongoing COVID-19 pandemic. New evidence suggests that, in addition to existing infection control standards, there is an urgent need for the incorporation of various recent information and advancements pertaining to structure (physical space, air handling, and BMW disposal) and process (human behavior, protocol compliance) to reduce self-contamination of HCWs during the doffing of PPE.
